# Synchronicity: The Role of Midbrain Dopamine in Whole-Brain Coordination

**DOI:** 10.1523/ENEURO.0345-18.2019

**Published:** 2019-05-03

**Authors:** Jeff A. Beeler, Jakob Kisbye Dreyer

**Affiliations:** 1 Queens College and the Graduate Center, City University New York, New York 11367, NY; 2 Department of Neuroscience and Pharmacology, University of Copenhagen, Copenhagen 2200, Denmark

**Keywords:** coherence, dopamine, phasic dopamine, striatum, synchronous dopamine activity

## Abstract

Midbrain dopamine seems to play an outsized role in motivated behavior and learning. Widely associated with mediating reward-related behavior, decision making, and learning, dopamine continues to generate controversies in the field. While many studies and theories focus on what dopamine cells encode, the question of how the midbrain derives the information it encodes is poorly understood and comparatively less addressed. Recent anatomical studies suggest greater diversity and complexity of afferent inputs than previously appreciated, requiring rethinking of prior models. Here, we elaborate a hypothesis that construes midbrain dopamine as implementing a Bayesian selector in which individual dopamine cells sample afferent activity across distributed brain substrates, comprising evidence to be evaluated on the extent to which stimuli in the on-going sensorimotor stream organizes distributed, parallel processing, reflecting implicit value. To effectively generate a temporally resolved phasic signal, a population of dopamine cells must exhibit synchronous activity. We argue that synchronous activity across a population of dopamine cells signals consensus across distributed afferent substrates, invigorating responding to recognized opportunities and facilitating further learning. In framing our hypothesis, we shift from the question of how value is computed to the broader question of how the brain achieves coordination across distributed, parallel processing. We posit the midbrain is part of an “axis of agency” in which the prefrontal cortex (PFC), basal ganglia (BGS), and midbrain form an axis mediating control, coordination, and consensus, respectively.

## Significance Statement

Dopamine is widely associated with providing value-related signals that serve activational and teaching functions. We shift focus from computing value-related signals and develop a hypothesis that suggest midbrain dopamine is sampling and integrating distributed neural activity to recognize and signal consensus across distributed, parallel processing. We posit each dopamine cell acts like an index of afferent activity and serves as a Bayesian unit detecting opportunity reflected in activity across distributed substrates. When cells across a dopamine population converge on the same temporal patterns, dopamine cell synchrony emerges generating a consensus signal that facilitates responding and learning. In this view, the fundamental role of dopamine is to signal consensus across parallel processing to facilitate unified behavioral responding.

## Introduction

The midbrain dopamine system is integral to mediating value-based neural activity underlying adaptive behavior (Wise, 2004; Schultz, 2007; Berridge, 2007; Salamone and Correa, 2012). Evidence continues to accumulate that midbrain dopamine can signal (1) obtained value, (2) value prediction, and (3) value prediction errors (Watabe-Uchida et al., 2017; Berke, 2018). Dopamine activity has been shown to be sufficient for mediating learned associations between stimuli and reward (Steinberg et al., 2013), demonstrating it can serve a crucial role as a value teaching signal (Schultz, 1998; Hart et al., 2014; Eshel et al., 2016; Kishida et al., 2016; Sharpe et al., 2017). Dopamine has been shown to modulate value-based behavioral choice and to increase (energize) responding to value predictive stimuli (Roitman, 2004; Hamid et al., 2016; Ko and Wanat, 2016; da Silva et al., 2018; Saunders et al., 2018). Dopamine has long been believed to provide a simple, scalar signal broadcast widely across the brain (Schultz, 1998; Bar-Gad et al., 2003; Joshua et al., 2009), essentially uniform across dopamine cells (Schultz, 1998; Eshel et al., 2016). Insofar as decision making is distributed across neural substrates, value must be mapped onto those distributed processes. By widely broadcasting a uniform, scalar signal, dopamine is commonly believed to deliver value information across neural substrates, mediating value-based modulation of processing and plasticity in target regions.

The nature of dopamine’s contribution to value-based adaptive behavior continues to be contentious. While evidence demonstrates that dopamine can encode prediction errors (Hart et al., 2014; Eshel et al., 2015; Watabe-Uchida et al., 2017), dopamine can signal value per se as well, described by Hamid et al. (2016; Berke, 2018) as an instantaneous value signal. Dopamine signals are not restricted to reward. Bromberg-Martin has proposed that dopamine can provide alerting, reward and aversive signals (Bromberg-Martin et al., 2010), although these can be construed as value related. Dopamine responds to novelty (Horvitz, 2000; Overton et al., 2014), although this can be construed as an “information bonus” (Kakade and Dayan, 2002). Dopamine signals have been linked to motor activity independent of reward (Pasquereau and Turner, 2015; Dodson et al., 2016; Howe and Dombeck, 2016). Other evidence suggests that moment-to-moment motivational states can modulate dopamine (Satoh et al., 2003; Syed et al., 2016). Dopamine prediction error signals are intimately linked to timing of events (Pasquereau and Turner, 2015; Takahashi et al., 2016; Starkweather et al., 2017; Coddington and Dudman, 2018; Langdon et al., 2018), and conversely, changes in dopamine have been demonstrated to affect timing (Soares et al., 2016). Dopamine has been demonstrated to play a role in arousal (Eban-Rothschild et al., 2016), suggesting an additional dimension in dopamine signaling. The notion that dopamine cells provide a uniform, broadly distributed signal is increasingly contested (Willuhn et al., 2012; Roeper, 2013; Lammel et al., 2014; Sanchez-Catalan et al., 2014; Morales and Margolis, 2017; Saunders et al., 2018), with data supporting both homogeneity across dopamine cell activity (Eshel et al., 2016) and diversity in dopamine signals in target regions (Matsumoto and Hikosaka, 2009; Lerner et al., 2015; Parker et al., 2016). In computational models, dopamine signals have been construed to represent abstracted value information divorced from details of stimuli and actions, i.e., “model-free” (Montague et al., 1996). It is becoming increasingly clear, however, that dopamine encodes and responds to features of stimuli and reward (Keiflin et al., 2019; Takahashi et al., 2017) and can be “model-based” (Daw et al., 2011; Daw, 2012; Langdon et al., 2018).

Many excellent reviews discuss the various issues outlined (Schultz et al., 2017; Berke, 2018; Watabe-Uchida et al., 2017). Here, we will describe an alternative view of dopamine function, shifting from the contentious question of what dopamine encodes to the comparatively less examined question of how dopamine encodes the information contained in its signals. Our hypothesis will posit that instead of serving an informational function of distributing reward-related information per se, dopamine can be construed as delivering a signal that serves to coordinate distributed, parallel processing by providing a “consensus” signal. We briefly review key characteristics of the midbrain dopamine system relevant to our hypothesis before tackling the task at hand.

## Current Ideas on Derivation of Dopamine Value-Related Signals: The Problem of Anatomy

How is the dopamine signal derived? Earlier models were based on variants of the algorithmic actor-critic model (Barto, 1995). These models presupposed dopamine to encode a prediction error and so the task was to determine how the quantities necessary for that computation (e.g., expected reward V_t_, obtained reward r_t_) were delivered to the midbrain. These models centered around the basal ganglia (BGS) connections with the midbrain (Houk et al., 1995; Brown et al., 1999; Contreras-Vidal and Schultz, 1999). While elegant, the models did not align with anatomy (Joel et al., 2002). Subsequent models incorporated additional anatomic features, such as the “spiraling” connectivity (Haber et al., 2000) of midbrain dopamine and striatal territories (Haruno and Kawato, 2006), a role for the pedunculopontine tegmental nucleus in value representations (Kawato and Samejima, 2007) and the pathway from striatal matrix projection neurons to dopamine cell dendrites in the substantia nigra reticulata (Tan and Bullock, 2008), or simply including more contributing regions (Vitay and Hamker, 2014). Hazy and colleagues built a model around substrates known to contribute to Pavlovian learning (Hazy et al., 2007, 2010). More recently, Eshel et al. (2015) proposed that GABAergic cells in the midbrain receive expected reward information that ramps up as reward approaches and drives local inhibition of dopamine cells, providing a simple subtractive mechanism for deriving a reward prediction error (RPE). Although elegant, the Eshel model begs the question of where the expected value signal in midbrain GABA cells is derived, effectively returning us to prior models with an added layer of complexity.

What every proposed model to date shares in common is the premise that discrete quantities, such as expected value and received reward, are computed in specific afferent regions and delivered to the midbrain to be used in computing value-related signals, typically construed as an RPE. However, this tidy compartmentalization and assignment of computational terms to discrete afferents is not supported by evidence. In recent years, a spate of elegant anatomic studies has reinvestigated the connectivity of midbrain dopamine using newer tracing methods, including tracing input/output relationships. In these studies, what stands out is the extent to which the inputs to the dopamine cells, regardless of their projection targets, arise widely from across the brain (Watabe-Uchida et al., 2012; Yetnikoff et al., 2014; Beier et al., 2015; Lerner et al., 2015; Carta et al., 2019). That is, it is the diversity and apparent non-specificity of afferent inputs that is most striking (Yetnikoff et al., 2014). How is this to be reconciled with the notion of discrete regions computing and delivering, for example, expected reward? What are the rest of the afferent inputs doing? A recent study by Tian et al. (2016) further highlights this problem. The authors recorded from different afferent inputs to the midbrain, as well as midbrain dopamine cells, and correlated firing patterns in afferent projections with task activities (cue, receiving reward, etc.). Rather than any clear segregation in afferent regions of specific quantities, reward, predicted value, error signals, they found that all of these quantities were distributed across the inputs tested with many regions and even individual cells showing mixed patterns of phasic activity (e.g., both cue and reward activation), seemingly belying any notion of tidy segregation of discrete quantities being computed in localized neural substrates and sent to the midbrain. It seems a little like everything is everywhere.

Aside from the diversity of inputs and the promiscuity of value-related afferent information, there is a more fundamental problem facing models that propose discrete quantities are computed in specific afferent regions and delivered to the midbrain. Most of the regions contributing afferents to the midbrain operate with ensemble encoding; that is, information is differentially encoded in various patterns of neural activity. Dopamine neurons, in contrast, are believed to act largely en masse and collectively encode a value-related signal delivered as a uniform scalar. How is a representation of a stimulus or action encoded in a patterned subset of cells (a CS+, for example) in a region such as the amygdala translated into a single quantity (e.g., expected value) and then distributed broadly across a population of dopamine cells? Short of every neuron in an ensemble within a particular afferent region being connected with every dopamine cell, it is not clear how this ensemble->scalar translation could be achieved and uniformly deliver the required quantity across the midbrain.

Together, these issues argue that the notion that discrete quantities are computed in particular afferent regions and delivered to the midbrain is not consistent with the anatomy and largely untenable. Instead, it appears that the midbrain is integrating a highly diverse set of inputs drawn broadly from across the brain and that these inputs may contain various, non-segregated value-related information. While this provokes the question of what information is being integrated, how and to what end, before considering this we have to consider the particularities of the output side of the dopamine system.

## The Necessity of Dopamine Cell Cooperation: The Problem of Synchrony

The midbrain dopamine nuclei contain a modest number of neurons, in rodents ∼20,000–40,000 tyrosine hydroxylase expressing neurons, estimated to be between 400,000 and 600,000 in humans (Björklund and Dunnett, 2007). Each neuron projects extensively across a large territory of target region(s). Individual SNC dopamine neurons are estimated to make 100,000–250,000 synapses in the rat and 1–2 million in humans (Bolam and Pissadaki, 2012), cover 6% of striatal volume and affect ∼75,000 medium spiny neurons (MSNs) (Matsuda et al., 2009). Conversely, each MSN synapses with a few hundred to over a thousand dopamine terminals (Arbuthnott and Wickens, 2007; Bolam and Pissadaki, 2012) and each MSN is estimated to be under the influence of 100–200 different dopamine neurons (Matsuda et al., 2009). This broad axonal distribution with overlapping spheres of influence is characteristic of volume transmission attributed to dopamine (Agnati et al., 1995; Zoli et al., 1998). Rather than releasing neurotransmitter discretely in a synapse, which then transmits an ultra-targeted intra-synaptic signal quickly terminated by reuptake, dopamine operates by modulating its extracellular concentration (Garris et al., 1994; Gonon, 1997; Cragg and Rice, 2004; Moss and Bolam, 2008; Dreyer et al., 2010). These characteristics of midbrain dopamine suggest that to effectively transmit a signal, dopamine cells must work cooperatively.

A dopamine signal encoded through modulation of cell spiking is decoded in target regions by modulation in receptor occupancy: thus, encoding-decoding fundamentally reflects a transformation from spike rate (frequency modulation) to fluctuations in extracellular dopamine concentration (amplitude modulation). While many factors can modulate this transformation, the critical determinant is the degree of synchrony among spiking neurons relative to the clearance rate of the dopamine transporter (Dreyer et al., 2010; Dreyer and Hounsgaard, 2013; Dreyer, 2014). When spike activity is correlated between neurons on a timescale similar to *K*_m_/*V*_max_ (corresponding to 100 ms in nucleus accumbens and 40 ms in dorsal striatum), release will integrate and generate temporally resolved peaks and troughs in extracellular dopamine that maximize information transfer to postsynaptic targets. When spike activity is asynchronous, including asynchronous bursting activity, dopamine concentration will be distributed across time, reflecting the average spike rate. We will refer to “tonic” and “phasic” in terms of whether population spiking activity generates temporally resolved fluctuations in extracellular dopamine (phasic, in phase) or a distributed averaging of activity (tonic, dopamine tone). Rather than reflecting two distinct modes of signaling, tonic and phasic represent two ends of a spectrum in temporal partitioning of dopamine cell activity. Phasic dopamine signaling has been construed as arising from uniformity in bursting activity, consistent with electrophysiological evidence where dopamine cell recordings show a high percentage o*f* tested cells exhibit similar and consistent responses to stimuli (Eshel et al., 2016), as well as with electrochemistry recordings of released dopamine, which fundamentally reflect the combined activity of hundreds of terminals (Dreyer et al., 2016).

While initially it was thought that dopamine cells are in some way coupled generating obligate synchrony, accumulating evidence favor emergent synchrony in which cells function autonomously, but independently come to generate the same temporal patterns of activity. Gap junctions between dopamine cells (Grace and Bunney, 1983) have been proposed to mediate uniformity in dopamine cell activity. These direct cell to cell connections may be less prevalent than originally thought, with estimates suggesting only ∼20–25% of dopamine cells are connected by gap junctions (Vandecasteele, 2005). These connections may provide a low-pass filter such that the activity in one neuron can influence firing rate in a connected neuron, but without transmitting yoked action potentials; no spike synchrony was observed in recorded pairs and mutual information was weak (Vandecasteele, 2005). There is little evidence that these gap junction connections induce an obligate synchrony, although such gap junctions could entrain network activity (Komendantov and Canavier, 2002) and may play a role in promoting emergent synchrony.

In studies examining pairs of dopamine cells, roughly 25% of dopamine cell pairs show significant correlation in spiking rate (Wilson et al., 1977; Hyland et al., 2002; Morris et al., 2004; Li et al., 2011) with little evidence of direct spike-to-spike correlation (Morris et al., 2004). The degree of correlation can be modulated by reward associated stimuli, learning and pharmacological manipulations. Using noise correlation analysis (correlation between two cells in their trial-to-trial variation from average response), Joshua et al. (2009) demonstrate that correlation in spike variability between dopamine cells increases with salient stimuli (cue, outcome). Kim et al. (2012) show an increase from 34% (noise) correlated cell pairs on initial exposure to a task to 49% and 66% after eight and 16 weeks of training, respectively. Finally, Li et al. (2011) demonstrated that application of nicotine increase the percentage of correlated dopamine cell pairs from 21% to 44%. These data suggest emergent rather than obligate synchrony. Many have previously suggested that synchronous dopamine cell bursting activity is necessary to generate temporally resolved extracellular dopamine signals in target regions (Venton et al., 2003; Arbuthnott and Wickens, 2007; Dreyer et al., 2010; Owesson-White et al., 2012), an idea we will place at the center of our hypothesis.

## Shifting Frameworks: From Value to Coordination of Distributed, Parallel Processing

We are presented then with a stark contrast between midbrain dopamine input, highly diverse afferent inputs drawn broadly from across brain regions in which value-related information does not appear to be neatly compartmentalized by region but rather widely distributed and intermixed, and the dopamine output system, which relies on relative uniformity of spiking activity across dopamine cell populations to transmit a simple scalar to target regions. It is this transformation from heterogeneity of input to homogeneity of output that will concern us: we propose the transformation from polyglot to monosyllable is fundamental to dopamine’s function within the brain, and that the emergence of dopamine cell synchrony is the core mechanism mediating this function.

Before describing our hypothesis, we want to tentatively reframe the putative problem the dopamine system evolved to solve. Widely viewed as the “reward transmitter,” various theories of dopamine describe a system that, in one way or another, helps the organism recognize and act on opportunities for value. In its most simplistic terms, the core evolutionary problem addressed is helping an organism to engage in actions that are advantageous, specifically by signaling value in some form (e.g., value per se, errors in value prediction).

We wish to consider dopamine in the context of a different core evolutionary problem. A fundamental question in neuroscience is how distributed, parallel processes are integrated into a functional “whole-brain” model to generate unitary organismal action (Breakspear, 2017; Christophel et al., 2017). The brain must process a continuous stream of on-going sensory information and from this parse actionable stimuli and emit advantageous responses, and do so rapidly (Cisek and Kalaska, 2010). Distributed, parallel processing facilitates rapid, efficient responding in the face of computational complexity. Each region/substrate forms its own, partial model of self-in-world. Simplistically, the hippocampus forms a spatial model: where actionable stimuli and events are located (Gauthier and Tank, 2018; Mamad et al., 2017; Stachenfeld et al., 2017). The amygdala forms a model of the valence of stimuli in relation to the organism (Beyeler et al., 2018; O’Neill et al., 2018; Pryce, 2018). The parietal cortex forms a model of egocentric sensorimotor space (Buneo and Andersen, 2006; Cui, 2014; Whitlock, 2014; Takiyama, 2015) and so on. Given that there is only one motor plant, these parallel models or representations must somehow be integrated into decision making that determines unitary organismal behavior, an organism cannot go right and left at the same time. Commonly construed as a simple scalar distributing value-related information, here we will entertain the possibility that dopamine provides a signal that coordinates rather than informs distributed, parallel processing, facilitating unitary self-in-world action from a multiplicity of neural representational models.

## Searching for the Ghost in the Machine: A Case for Implicit Value

The function broadly associated with dopamine involves recognizing available reward opportunities, weighting decision making to act on opportunity, energizing responding and facilitating learning about the context and predictors in which the reward occurred: in short, to facilitate adaptive choices in the face of opportunity. Is this not what the entire brain evolved to do? “Value” is a (perhaps the) fundamental organizing principle of the brain. It is not merely that value is reflected in on-going neural processing broadly across neural substrates (e.g., primary visual cortex, hippocampus) but that the brain is structurally shaped and modified by value. During early development, synapses are pruned to select those that yield functionality (i.e., value) to the organism. Throughout life, neural plasticity shapes circuits and weights synapses to benefit the organism, i.e., obtain value.

The challenge is ascertaining the root of the evaluative mechanisms that guide this value organization. How does the brain know what is good for it? While there are many potential answers to this question, one common answer is that dopamine distributes value information. Indeed, that is the core role of the reward transmitter regardless of one’s view on the form in which this information is delivered. But this immediately begs the question “how does midbrain dopamine ‘know’?” Who teaches the teacher? That is, how is this signal derived? All extant theories suggest that the midbrain obtains value information from afferent regions, but this quickly becomes circular. If dopamine is the teacher, how do afferent regions learn about value to inform the midbrain, and if afferent regions already recognize value, what need is there for a teaching signal in the first place? In short, the notion that dopamine distributes value information across the brain begs the question of how and where value is originally determined. Where is the ghost in the machine?

While the brain must select stimuli to which to direct its attention and generate advantageous responses, the most fundamental selection process is how the brain represents self and world in the first place. That is, it is neural activity itself that must be selected to create adaptive, productive self-in-world models that, in turn, yield adaptive behavior. This assertion does not resolve the question posed above about how value is determined, but simply restates it in neural terms. However, doing so provides a different perspective. Rather than construe value as some quantity computed somewhere, we view the brain as being in a constant process of selecting productive and diminishing non-productive neural activity. While we cannot pretend to explain how this happens, we do not know where the “ghost” resides, we can draw a tentative corollary: value is implicit in neural activity. While this may sound radical, it is consistent not only with a broad construal of the fundamental function of the brain in mediating adaptive behavior, it is consistent with empirical studies that tend to find value information, in a variety of forms, distributed broadly across the entire brain (e.g., Tian et al., 2016, but many others). From here we will sketch out our hypothesis of midbrain dopamine function, building around the core ideas that dopamine is (1) integrating diverse afferent activity that reflects implicit value and (2) generating a scalar output signal when this value information from different afferents temporally converges signaling “agreement,” or consensus across distributed, parallel processing, that something important is happening now; that is, the whole brain is in agreement, here is an opportunity, time to act.

## Reconceptualizing Midbrain Dopamine as Sampling-Distributed Activity across the Brain

**Figure 1.**
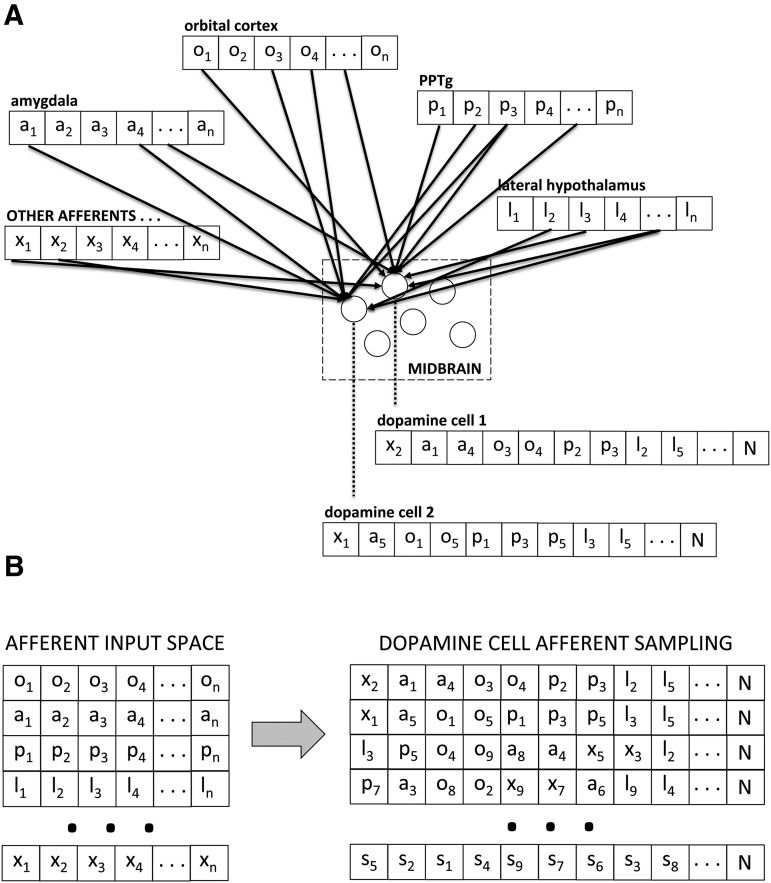
Random distribution of afferents to dopamine cells as sampling the input space. ***A***, Projections from each afferent region construed as a vector randomly distributed to individual dopamine cells. ***B***, Afferent projections to midbrain comprise a total vectorized input space from which individual dopamine cells represent random samples of that space.

Watabe-Uchida et al. (2012) note that afferents into the midbrain, rather than reflecting distinct connectivity between functional brain regions seems to arise in diagonal bands across the brain that do not respect functional regions. Rather than try to find order in afferent connectivity to the midbrain, we propose the midbrain randomly samples activity broadly across the brain and functions as an index of that activity, much in the way that a sample of stocks in the stock market serves as an index of the market. Consider the axons streaming from each afferent region to the midbrain as a vector where each axon delivering a continuous, rate-based signal is an element in the vector and the vector represents the total output to the midbrain from that afferent region. We posit that these axons, each an element in the afferent vector, are distributed randomly to dopamine neurons (Fig. 1*A*). If each dopamine neuron is, in turn, viewed as a vector composed of its various inputs, each dopamine cell represents a random sample of afferent activity where the afferent vectors from input regions collectively comprise a multidimensional space (Fig. 1*B*). We construe this space as event driven, i.e., that neural activity is continuous and constantly responding to on-going sensorimotor information. An event such as a cue-light, then, would be represented in this multidimensional space as a composite of the vectors representing individual afferent regions. Stimuli, actions, events, memories, everything encoded by the brain, would generate a unique composite in this multidimensional space.

Each individual dopamine cell represents a random sample of this multidimensional space. In inferential statistics, we use probability theory to ask “how likely is it that this difference arose as chance and if I repeated the study over and over would this effect persist?” An alternative strategy, although impractical, would be simply to simultaneously replicate a study thousands or millions of times always with a new randomly drawn sample. So, we argue, with dopamine. A single dopamine cell may increase its firing in response to increased afferent activity, but this could arise spuriously from the particular randomly distributed afferents it receives without reflecting something important arising in the multidimensional input space overall, such as a cue-light that comes to organize neural activity across multiple brain regions. Fortuitously, when a single dopamine cell starts bursting independent of the population, it will have little effect on output at target regions. Indeed, phasic activity is not a rare event correlated only with value. Dopamine cells continuously intermix tonic and phasic activity (Marinelli and McCutcheon, 2014) with dopamine output determined by population activity (Dreyer et al., 2010); that is, when they fire synchronously. Thus, the 40,000 dopamine cells in the rat midbrain could be construed as 40,000 random samples, 40,000 “studies” occurring concurrently, and when a large percentage of them are driven to burst firing simultaneously, this generates a temporally resolved dopamine signal in target regions that carries value-related information. Because this multi-dimensional afferent input space is comprised of activity arising in parallel, distributed processing, for a stimulus such as a cue-light to generate population-based, synchronous phasic activity in midbrain dopamine, the stimulus must presumably have a widespread effect across neural regions. Because of this, we construe the emergence of temporally resolved phasic dopamine signals that arise from synchronous bursting activity as signaling consensus, a consensus across afferent regions about the importance of on-going sensorimotor information being processed in parallel across distributed substrates.

## Axis of Agency: Sketching a Broader Hypothesis of Coordination across Distributed Processing

Our purpose here is to initially formulate a novel hypothesis of dopamine function. Because we shift our framework from the problem of computing and delivering value information to the problem of facilitating coordination across distributed, parallel processing, we must necessarily put our discussion of dopamine into this broader context and provide a rough sketch of how the brain achieves coordinated distributed processing, in which we posit the midbrain plays a specific role.

**Figure 2.**
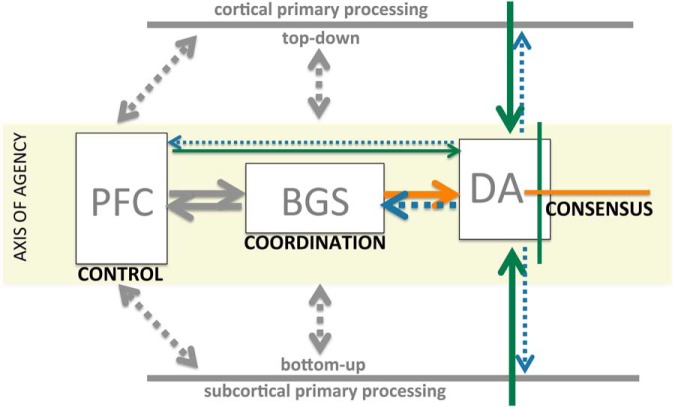
Schematic of “axis of agency.” Primary processing substrates (distributed, parallel processing) represented abstractly in gray as either top-down or bottom-up substrates with reciprocal connections to the prefrontal cortex (PFC) and basal ganglia system (BGS), not detailed here. The three conceptual nodes in the axis of agency are indicated in boxes with their hypothetical role in mediating coordinated activity across distributed substrates noted in bold below. Only the inputs/outputs of dopamine, the focus of this perspective, are colored, with green and orange representing excitatory and inhibitory inputs and blue dopamine outputs. The role of the BGS and midbrain dopamine is elaborated below.

We posit four levels of substrate in describing coordination across distributed processing (Fig. 2). We posit four levels of substrate in describing coordination across distributed processing. The first is primary processing in individual neural regions, such as the amygdala, hippocampus and so on. Although these are intricately interconnected (e.g., frontal and parietal regions), each region specializes in capturing some aspect of an overall model of self-in-world. A second substrate level would be where disparate processing converges onto a substrate that does not have its own assigned task but is assigned to ensure that the work of the various parallel processing does not begin to conflict or go in different directions viewed collectively as a whole. We posit that the BGS serve this function (elaborated below), which we describe as integrative discriminative selection. “Integrative” because they integrate disparate inputs from across distributed processing and “discriminative selection” because, in turn, they modulate efferent targets (generally the same regions providing afferents) by selecting which activities to facilitate and which to inhibit (selection), doing so by discriminating which afferent inputs facilitate positive outcomes and which do not. This substrate can effectively modulate the activity of individual primary substrates “in the context” of other primary substrates: integrative discriminative selection. Third, we posit a signal that indicates when there is agreement across distributed processing to mobilize action and facilitate learning, a function we attribute to midbrain dopamine. We would describe this function as integrative consensus: integrative because multiple, diverse inputs are being integrated in a polling manner (“sampled”) and consensus because the goal is to determine when something appears in the on-going sensorimotor stream that is recognized across multiple substrates as being of value and broadcasting a signal when this occurs. Finally, there needs to be some primary control structure that steers this “ship of coordination,” which we attribute to the prefrontal cortex (PFC).

We will briefly describe this view of the BGS as it is central to elaborating our hypothesis of dopamine. It is difficult to cogently discuss the function of either dopamine or the BGS without taking both in account as they are densely interconnected (Watabe-Uchida et al., 2012; Guo et al., 2015) and their respective functions profoundly intertwined. We will not discuss primary processing or associated interconnectivity nor will we elaborate on the role of the PFC as “steering the ship” as much has already been written on the PFC as an executive locus.

## The BGS: Selection of Composite Representations

The primary input nucleus of the BGS, the striatum, has been thought to be an integrative substrate for decades (Hikosaka et al., 2014; Kemp and Powell, 1971). Processing convergent information from multiple sources, the BGS in turn modulate the regions that provide afferent input, illustrated best by the cortico-BGS-cortical loops. Considered in the context of facilitating coordination across distributed, parallel processing, we propose that the BGS function analogous to a hidden layer in neural networks. Inputs are distributed across units in a combinatorial fashion allowing the discriminative selection of those combinatorial units associated with positive outcomes. Across learning, this combinatorial selection process progressively modulates those same brain regions that provide afferent input. Because the striatum is integrating information from across distributed, parallel processing into these combinatorial units, this provides a substrate in which activity in one region can be modulated “in the context of” other regions. We elaborate this briefly.

### The mosaic: substrate for combinatorial integration and selection

Da Cunha et al. (2009) liken the striatum to a “mosaic of broken mirrors.” This is to say that cortical representations are not mirrored directly in the striatum but are broken up, with fragments of cortical representations distributed broadly across striatal MSNs. Each MSNs, in turn, receives fragments of cortical representations in a combinatorial manner. Thus, discrete cortical representations become fragmented and intermixed: mosaic of broken mirrors. This is consistent with known anatomy. A single MSNs receives ∼5000 cortical afferents (Alexander, 1994), although individual cortical axons will make only one to two contacts with a single MSNs (Tepper et al., 2007), suggesting both tremendous distribution and convergence. Conversely, a single cortical axon can innervate up to 14% of the striatum (Zheng and Wilson, 2002). While the striatum has been associated with dimension reduction (Bar-Gad et al., 2003), this fragmentation and expansion of cortical representations into combinatorial units (the mosaic) could be view as dimension enhancement. Introducing combinatorial dimensions allows a more expansive and powerful substrate for discriminative selection compared to faithfully recapitulating or mirroring cortical representations discretely in the striatum.

### At the crossroads: integrating distributed, parallel processing in selection

Noting that the inputs from multiple afferent regions (amygdala, hippocampus, PFC) intersect in the ventral striatum, Humphries and Prescott (2010) characterized this intersection as a “crossroads” where the activity from disparate regions come together and interact, integrating their activities in the output of the ventral striatum. This convergence of afferents from disparate regions is observed across the striatum (Choi et al., 2017). Building on the mosaic idea above of expanded, fragmented and distributed cortical representations, the crossroads notion provides a basis whereby the selection and modulation of these fragmented cortical representations can be modulated by inputs from other regions, providing a substrate where distributed parallel processing across multiple afferent regions are integrated into a combinatorial “mosaic” in which what is being selected is not discrete actions or stimuli per se, but combinations of neural activity across distributed substrates that reflect parallel processing of stimuli and actions: a composite representation. In essence, the BGS select when concurrent activity across distributed substrates is productive and facilitates that concurrent activity in target/afferent regions.

### A little bit of everything, everywhere

While most brain regions have been associated with particular kinds of information, the striatum exhibits remarkable malleability in its encoding. In virtually every task in which striatal activity has been monitored, every aspect of the task has been represented: stimuli/cues, actions, rewards as well as putative predicted value associated with these representations. Moreover, medium-spiny neurons will come to represent whatever is relevant in a task. If spatial locations are relevant, spatial locations will be represented in phasic MSN activity (Shibata et al., 2001; Chang et al., 2002). If time is important, time will be represented (Taha et al., 2007; Day et al., 2011). In short, across learning the striatum appears to form a representation of any task where salient aspects of the task are differentially represented in phasic activity in subsets of MSNs. This malleability is consistent with the notion of discriminative selection on a combinatorial substrate integrating disparate afferents, as we propose.

### Different kinds of integration

In our characterization of the BGS and midbrain dopamine, we suggest both are integrating disparate afferents drawn broadly from across the brain, but the function of this integration is essentially opposite. During learning, dopamine cell activity becomes increasingly synchronous and homogeneous across a population of dopamine cells (Eshel et al., 2016). In contrast, MSNs develop differential patterns of phasic activity in response to discrete stimuli and task events (Tremblay et al., 1998; Hikosaka et al., 1989a, b,c; Nicola et al., 2004; Day et al., 2006; German and Fields, 2007), suggesting discriminative learning. In short, while dopamine cells in a population converge in their signaling, striatal cells diverge, facilitating a discriminative selection process.

### Striatal composite representation modulates distributed processing

Gerraty et al. (2018) demonstrate that learning to associate a visual cue with reward involves dynamic changes in the coupling between striatum and multiple brain regions, including frontal and visual cortices, and that these changes are correlated with learning rate (see also den Ouden et al., 2010; van Schouwenburg et al., 2010; Horga et al., 2015). Thus, returning to our larger perspective of assigning the BGS a role in mediating coordinated activity across distributed, parallel processing, we suggest that the BGS, via the striatum, create a mosaic or combinatorial composite of activity from multiple, disparate neural regions (integrative) and use this composite substrate to discriminatively select those combinatorial units (analogous to hidden units) that are associated with positive outcomes to broadly modulate distributed processing across the brain, facilitating coordination across distributed processing that is advantageous to the organism. A critical aspect of this function is that it operates via disinhibition; that is, the BGS exert a tonic inhibition that is selectively released, an operational characteristic we will further build on below. A central requirement for this proposed striatal/BGS function is some mechanism by which selection is guided, a teaching signal. Dopamine has long been believed to provide this teaching signal (Schultz et al., 1997; Schultz, 1998; Wise, 2004), returning us to midbrain dopamine.

## Midbrain Consensus Signaling: Emergent Synchrony in Dopamine Cell Activity as a Bayesian Selector

**Figure 3.**
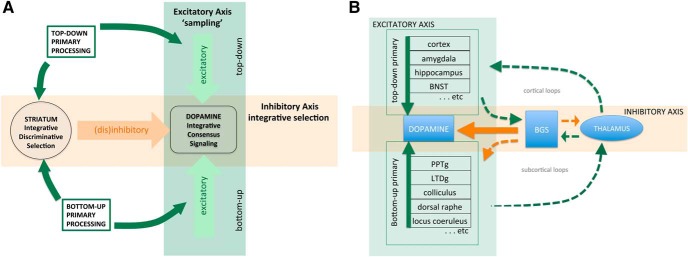
Two axes model of dopamine integrative consensus signaling. ***A***, Overall conceptual rendering of proposed model where midbrain dopamine integrates two primary axes of input, (1) a (dis)inhibitory axis arising from the BGS (ventral pallidum, striosomes, accumbens) and (2) a largely excitatory axis arising from distributed afferents across the brain, reflecting both top-down (e.g., cortical inputs, amygdala, hippocampus, BNST) and bottom-up information processing (e.g., collicular, multiple brainstem afferents). ***B***, A more anatomic rendering incorporating cortical and subcortical loops through the BGS. For detailed cataloging of dopamine inputs, see Watabe-Uchida et al. (2012), Lerner et al. (2015), and Beier et al. (2015). Basal ganglia system (BGS), bed nucleus of stria terminalis (BNST), pedunculopontine tegmental nucleus, (PPTg), laterodorsal tegmental nucleus (LTDg).

We propose that midbrain dopamine functions at the intersection of two axes (Fig. 3): (1) a largely excitatory axis “sampling” distributed, parallel activity that drives dopamine activity, particularly bursting and (2) an inhibitory axis derived from the striatum that gates dopamine cells as a function of striatal integrative discriminative selection process we described above. We propose that these two axes can be construed as “advocate” and “skeptic” in implementing a Bayesian selection process at the level of individual dopamine cells. Congruence in these two axes, that is, excitatory drive is complemented by selective disinhibition, facilitates increased dopamine cell activity. When this arises across a large percentage of dopamine cells, a product of learning, synchrony emerges facilitating a population based phasic signal.

**Figure 4.**
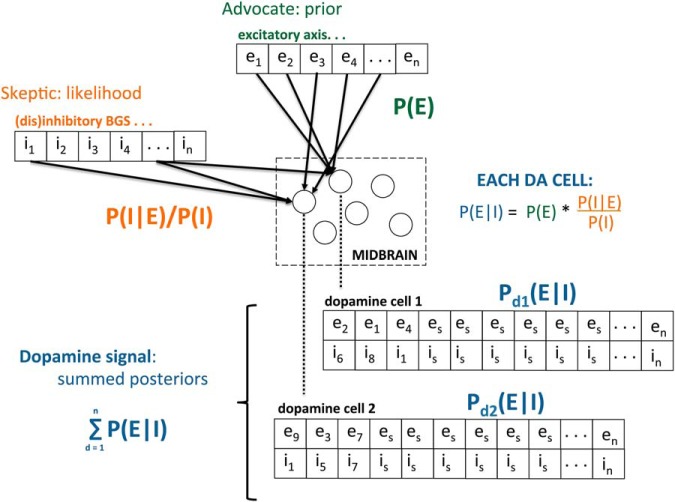
Illustration of midbrain dopamine as a Bayesian selector. Random distribution of vectorized inputs from an afferent input space, as illustrated in Figure 1, where each dopamine cell samples on-going activity, mapped onto a Bayesian construal. The excitatory axis (green) is assigned as the prior (the advocate) and the (dis)inhibitory axis (orange) is assigned as the likelihood (the skeptic). The posterior (blue) arises from the integration of these two axes (i.e., Fig. 3) at both the level of individual dopamine cells (firing rate) and at a population level, where synchrony determines the degree to which increases in firing rates in individual cells sum to produce a population-based phasic signal, which we construe as a consensus index, both consensus across dopamine cells as Bayesian units and consensus across the sampled input space, reflecting widespread afferent activity in response to current stimuli.

In our Bayesian construal, the computational goal is to determine the probability, the posterior, that distributed, parallel processes are responding to the same events in the on-going sensorimotor stream (e.g., a cue light predicting reward or the absence of an expected reward), reflecting the ability of those events to broadly organize neural activity, reflecting in turn implicit value; that is, to determine the probability that disparate primary substrates are organizing around and responding to the same events, consensus. Taking the excitatory (E) and inhibitory (I) axes proposed above as advocate and skeptic, respectively, we formulate our Bayesian construal as follows (Fig. 4):

The prior is comprised of the excitatory drive, P(E), reflecting the sampling of distributed processing discussed above. This afferent activity reflects “prior belief” because, as events enter the sensorimotor stream, the response of distributed afferents is shaped by prior experience and learning (response to novel stimuli addressed below). Thus, when multiple afferent regions respond to a sensorimotor event, this increases P(E), reflecting the prior probability that this event has an organizing impact on distributed processing.

The likelihood, P(I | E)/P(I), is a function of striatal inhibition of dopamine. The denominator, P(I) reflects tonic inhibitory tone; this is the skeptic, maintaining a basal, inhibitory non-belief that diminishes the excitatory prior. This basal inhibitory tone is modulated by afferent input to the striatum (from many of the same regions contributing to the midbrain), P(I | E); that is, striatal inhibitory tone given the excitatory drive on the striatum. Thus, as events in the sensorimotor stream, such as a cue light, disinhibit striatal inhibition of the midbrain, the skeptic is diminished granting greater weight to the prior. We note that the “E” in P(E) and P(I | E) reflect the excitatory drives on the midbrain and striatum, respectively, and are not strictly the same quantity. We construe them as serving the same function in our construal, reflecting activity across distributed, parallel processing, i.e., primary processing, funneled to both the striatum and midbrain for different purposes.

We propose that this Bayesian selector operates both at the level of individual cells and the population level. We suggested above that each dopamine cell is sampling afferent activity and serving as an index; here we elaborate this as a Bayesian index. As greater numbers of individual dopamine cells respond to the same events, emergent synchrony will increase, and with it a temporally defined phasic dopamine signal. Thus, the selector operates at the population level simply as a summation across cells, an index of indices, the sum of posterior probabilities from a population of afferent samples.

While we present here apparent mathematical quantities, P(E), etc., these are very different from discrete quantities proposed in prior models, such as expected reward, V_t_. P(E) can be construed as evidentiary without constraining the representational content of the afferent activity. Such activity, as Tian et al. (2016) demonstrated, can reflect value, prediction, prediction errors or, more to the point, activity arising entirely independent of value as an abstract quantity, such as signals reflecting proprioceptive or motor activity, motivational state/choice, arousal and so on. That is, as “evidence in favor of prior belief” the representational content of afferent input can be broad rather than constrained to a single quantitative representation such as value or prediction error. This is an initial conceptual sketch. How such Bayesian operations could be implemented at the cellular level would require further formal theoretical development and empirical testing of the hypothesis (for the challenges inherent in developing neural implementations, see Potjans et al., 2011, 2009).

In sum, we propose that midbrain dopamine is constantly sampling distributed neural activity monitoring for occasions when the input from both the advocate, P(E) and the skeptic, P(I | E)/P(I) agree that the current activity distributed across the brain reflects coordinated neural activity in response to an event in the sensorimotor stream that organizes distributed, parallel processing, reflecting implicit value: and as such, likely an opportunity to be acted on and learned about. When a large percentage of dopamine cells arrive at the same “conclusion,” synchrony arises and a temporally resolved fluctuation in extracellular dopamine emerges and encodes opportunity to act and learn.

## Distributed Cascading Learning: Midbrain Dopamine, the Last to Learn

**Figure 5.**
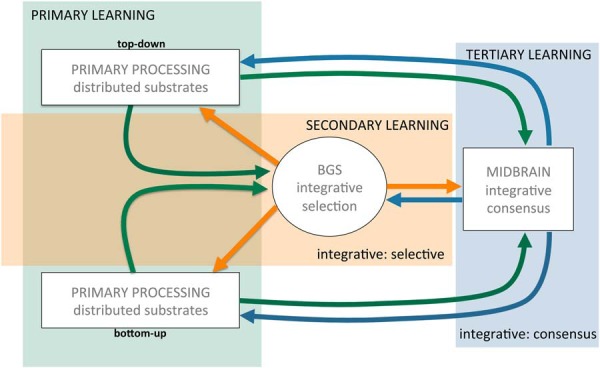
Cascading learning. Learning occurs progressively interleaved at three levels: (1) in primary models of individual afferent substrates, both top-down and bottom-up (blue), (2) in a secondary, integrative selection model in the basal ganglia system (BGS, orange), and (3) in the midbrain dopamine system itself (green). Arrows indicate how learning at different levels influences learning at other levels.

The notion that phasic dopamine signals consensus may at first seem contradictory to the widely accepted idea that dopamine provides a teaching signal. In our hypothesis, it appears that midbrain dopamine is the last to learn, not the teacher. This is consistent with studies that observe dopamine prediction errors emerge following, not preceding learning (Coddington and Dudman, 2018). We will briefly describe a whole-brain learning scheme in which learning occurs in a cascading, interleaved fashion along three axes (Fig. 5).

First, primary learning occurs in afferent regions contributing to midbrain dopamine, such as the PFC, amygdala, hippocampus; that is, across the distributed substrates we called primary processing and identify as the prior, P(E). Second, learning occurs in the striatum and BGS system, altering inhibitory drive on dopamine, the likelihood, P(I | E)/P(I). Finally, learning occurs in the midbrain itself via synaptic plasticity, selectively strengthening and weakening particular synaptic inputs. We view this learning as cascading because it occurs in an interleaved fashion where each axis of learning modulates the others. For example, as primary substrates undergo learning, this alters the input driving both the BGS and midbrain, modulating both P(E) and P(I | E) in our Bayesian construal. Learning and activity in the primary substrates are, in turn, modulated by both BGS output and dopamine signals. Similarly, learning in the BGS alters inhibitory input to the midbrain, and modulated dopamine in turn alters learning in the striatum. The core idea here is that learning does not occur discretely in one substrate and then get transferred to another as a teaching signal, but that learning occurs simultaneously across all substrates in an interdependent fashion.

A crucial objective for learning in distributed substrates is to coalesce into an organized whole-brain model to facilitate unitary action. The interleaving of BGS and midbrain learning contributes to the development of this coordinated organization. Midbrain dopamine signals growing consensus as learning progresses, arising through emergent synchrony (Kim et al., 2012), increasing agreement across dopamine cells in their Bayesian computations, which facilitates and accelerates learning as well as mobilizes resources and responding. The ability of midbrain dopamine to modulate learning and responding is effectively titrated by the degree of synchrony that contributes to a temporally resolved phasic signal. In this sense, dopamine has its greatest impact when it is maximally reflecting consensus. That is, the emergence of a phasic dopamine signals reflects the cumulative, convergent effects of cascading learning broadly distributed across the brain, consistent with observations that changes in dopamine signals can lag behind adaptive behavior (Coddington and Dudman, 2018).

This provides an alternative perspective on the emergence of prediction errors. Learning is initiated when an animal encounters known value (e.g., food pellet) at an unexpected place and time. The prediction error hypothesis argues that surprise, the unexpectedness of that initial encounter, generates the dopamine teaching signal. Our hypothesis focuses on the known value aspect of that initial encounter: the encountered food pellet is well known to distributed neural substrates and induces a consistent, organizing response in those substrates that drives a dopamine consensus signal to facilitate further learning about the features of time and place that yielded the food opportunity, such as a cue light predicting the reward. We would further suggest that the emergent phasic dopamine response to the cue light does not reflect dopamine teaching target regions about the value of the cue light but, rather, that the “rest of the brain” has learned the value of the cue light and, as the cue light becomes a stimulus of “known value” across the brain, dopamine now signals consensus regarding the cue light: the last to learn.

## Predicted Rewards and Novel Stimuli

We wish to briefly touch on two possible objections to our notion that dopamine signals consensus. In one, a lack of phasic dopamine can be observed when one might, according to our hypothesis, expect it. In the other, conversely, dopamine cell bursting is observed when there would seem to be no basis for consensus across distributed processing.

First, sometimes a well-predicted reward does not generate a phasic dopamine response. If the reward induces an organized, consistent response across distributed substrates, consensus, why does the dopamine signal disappear? We note first that this is not always the case, perhaps mostly not the case, and that a persistent dopamine signal in response to reward, even in well-learned tasks, has been repeatedly observed (Hamid et al., 2016, and many others). Nevertheless, when the loss of phasic dopamine in response to reward is observed, this arises when a task is overlearned. We would argue that this reflects automation where discrete stimuli (e.g., cue-response-reward) are “chunked” into a sequence as a single entity (Graybiel, 1998), where only the beginning of the sequence generates a phasic dopamine response, unless the outcome varies from how the sequence is expected to end. That is, the value of the reward is absorbed into the sequence rather than treated as a distinct event. In a sense, this is the same as saying the reward is predicted, only the proposed mechanism is different. Rather than a computation subtracting expected and received reward generating an error of zero when the reward is perfectly predicted, our hypothesis would argue that once a sequence of events (stimuli, actions, outcomes) have become automatized, the individual steps in the sequence no longer organize distributed processing nor drive the dopamine response; essentially, they are taken for granted until something goes awry, bringing non-automatic distributed processing back online to process the error, modulating dopamine.

Second, novelty is known to induce increased dopamine cell activity (Horvitz, 2000; Overton et al., 2014), which seems inconsistent with the notion that dopamine is signaling consensus: how can there be consensus across substrates with a newly encountered stimulus or context? Novel sensory information entering the brain does not have a learned path of propagation determined through prior experience; instead, we assume the novel information generates widespread activity across regions as the brain tries to process this new encounter based on prior experience and knowledge, with features of new stimuli partially activating memory of previous stimuli encountered as the brain uses its existing models to learn about the unfamiliar. We would expect this increased activity across distributed brain regions to drive the excitatory axis and generate bursting and small dopamine transients; however, we would not expect synchronous firing phase-locked with stimuli but randomly distributed bursts/transients throughout exploration and examination of novel stimuli. That is, this increased activity does not generate a temporally resolved signal arising from synchronous dopamine activity but instead the bursts/transients are distributed across time, effectively increasing tonic dopamine facilitating increased activity, exploration and plasticity. If new stimuli are repeatedly paired with value, they will come to evoke a clear, temporally resolved phasic signal arising from synchronous activity across a population of dopamine cells. If not, the animal will habituate and tonic dopamine will return to basal levels without acquiring a phasic response. In both cases, our arguments could be elaborated and refined, but space preclude exhaustive discussion.

## So, What Does Dopamine Encode?

Temporal-difference theories of dopamine have been largely based on the idea that dopamine signals arise from a model-free system (Montague et al., 1996). The values associated with a state (e.g., “cue light on”) are derived from experience in which the basis of that value is inaccessible, i.e., there is no model of how that value was derived, sometimes called a “cached” value. As a consequence, dopamine provides a “pure” value-related signal in a “common currency” that is independent of distinct sensory features of experience; that is, two rewards of equal value should be substitutable and generate the same dopamine signal. However, it has become increasingly clear that dopamine does not provide a pure, model-free signal of value (Takahashi et al., 2017; Keiflin et al., 2019); rather, it appears that dopamine signaling may be model-based (Daw et al., 2011; Daw, 2012; Langdon et al., 2018) such that not only changes in value, but the identity and characteristics of sensory information associated with that value can modulate the signal. Moreover, evidence is accumulating that midbrain dopamine may not be restricted solely to value-related signals. As discussed in the introduction, phasic dopamine activity has been shown to correlate to motor movement (Dodson et al., 2016; Howe and Dombeck, 2016), arousal (Eban-Rothschild et al., 2016), motivational state (Satoh et al., 2003; Syed et al., 2016), and timing (Pasquereau and Turner, 2015; Soares et al., 2016). How do we account for this apparent diversity in the “content” of dopamine signals, i.e., that dopamine does not correlate only to abstract value but can be correlated with a multiplicity of phenomena? Our hypothesis suggests the following.

### Primacy of temporal encoding

We suggest the crucial characteristic of dopamine signaling is when, not what. Dopamine mobilizes and energizes responding and facilitates learning. As a consensus signal, phasic dopamine is effectively saying “now,” which serves to activate and coordinate diverse target regions to collectively respond rapidly and vigorously in order to seize upon an opportunity. The crucial question, when is now?, can be answered with varying degrees of temporal precision: from a cue light indicating reward in one second to contextual stimuli, such as being in an task environment with a higher rate of reward availability (Howe et al., 2013; Hamid et al., 2016; Beeler and Mourra, 2018). In our consensus signaling theory, the precision of when is determined by the degree of synchrony among dopamine neurons, such that high synchronous activity leads to temporally resolved phasic signaling while asynchronous activity, even if bursting, leads to increased dopamine distributed across a period of time, i.e., increased tonic, as might occur in a high reward context. That timing is an integral part of dopamine signaling is an established idea, particularly with prediction error theories (Pasquereau and Turner, 2015; Soares et al., 2016; Starkweather et al., 2017; Langdon et al., 2018). Here, we suggest temporal characteristics are more than a feature of dopamine signaling, but its fundamental, primary function: the conductor’s baton signaling to distributed processing “this we know, act now.”

### Bayesian model of opportunity

The point above begs the question, temporal model of what? We argue the “what” is when an event in the sensorimotor stream impacts neural activity and processing across multiple afferent substrates indicating the event has an organizing effect on distributed, parallel processing, reflecting implicit value. Such implicit value may correspond to the traditional economic sense, such as when a cue light predicts the imminent arrival of a delectable, tasty treat, but may also reflect implicit value in stimuli that organize neural activity to achieve non-traditional value, such as the ability to run the rotarod without falling off, or to execute a killer tennis serve. In essence, it is the neural activity itself that is being assigned a valuation for its utility in achieving some goal rather than valuation of what it is that neural activity is putatively representing. In short, the what corresponds to “when this neural activity arises, it reflects an opportunity for advantage and should be promoted and acted upon.”

### Representational content embedded in dopamine signal is unconstrained

In our view, dopamine cells are collecting evidence, the midbrain is an evidentiary system, and that the representational nature (the content) of that evidence is unconstrained: basically, it can be anything and reflect whatever representational content is being processed in afferent regions driving midbrain dopamine activity. In our metaphor of a dopamine cell as analogous to an index of stocks, if one stock has a disproportionate effect on the index, say an automobile company, this does not mean that the index is “signaling automobile,” but if you look at what the index corresponds to in economic activity, it will correspond to increased car sales. Similarly, midbrain dopamine may generate a phasic response, signaling consensus and implicit value, that may correlate with any number of events, stimuli and actions depending upon the task. During a motor task such as the rotarod, dopamine cells may correlate to proprioceptive, vestibular and motor activity. During a task with visual cues, visual information may be embedded in and even dominate dopamine cell activity while in a task with auditory cues, auditory information dominates. In short, rather than encoding an abstracted value associated with different events, stimuli and actions, we propose that these appear in dopamine cell activity precisely to the extent to which they organize distributed, parallel processing in afferent regions, reflecting implicit not abstracted value.

## Areas for Further Development and Research

As noted above, many aspects of our hypothesis are consistent with extant data, including heterogeneity of afferents from distributed brain regions, the requirement for dopamine cells to fire synchronously to generate a temporally resolved phasic signal, evidence that dopamine cell synchrony can increase with learning, evidence that various types of value information are mixed and distributed across multiple afferent substrates, the mixed nature of the dopamine value signals (value, prediction errors), as well as accumulating evidence that dopamine signals can correlate to diverse phenomena beyond abstracted value (i.e., are multifaceted or multiplexed), including both features of stimuli associated with value (model-based signals) as well as “non-value” related activity, such as observed in non-reward related tasks (e.g., motor tasks). What we offer is a different framework in which to interpret these data and an alternative hypothesis on what the “basic function” of dopamine might be, shifting from signaling and teaching about reward or value to mediating coordination across distributed, parallel processing.

This initial description of our hypothesis, laying out the basic ideas and claims, lacks the rigor of a formal theory, which will emerge over time as the ideas presented are further developed. Nonetheless, the hypothesis does yield many predictions that can be tested empirically. For example, the notion that “dopamine is the last to learn” can be tested in a design similar to Tian et al. (2016), except looking at the progression of the mixed value signals in afferents compared to midbrain dopamine across learning. Similarly, optical tools can facilitate comparing inhibitory striatal inputs, P(I | E)/P(I), to excitatory inputs, P(E) to begin to empirically test and dissect our Bayesian formulation. Readers can discern such testable predictions themselves. Given the nascent nature of our hypothesis, we use available space here to note some limitations in our initial discussion and areas for further development, consideration and research.

### Limitations

First, it is becoming increasingly evident that both dopamine and midbrain GABA neurons form a functional midbrain unit (Brown et al., 2012; van Zessen et al., 2012), including the recent hypothesis that the dopamine prediction error arises as a subtractive computation between GABA and dopamine cells in the midbrain (Eshel et al., 2015). A more comprehensive hypothesis of “midbrain dopamine” may require inclusion of GABA neurons. Second, we have glossed over the extent to which midbrain dopamine may consist of functionally separate subpopulations that signal independently, possibly through different “channels” with distinct, segregated projection targets (Willuhn et al., 2012; Roeper, 2013; Lammel et al., 2014; Sanchez-Catalan et al., 2014; Dreyer et al., 2016; Morales and Margolis, 2017). Third, our hypothesis would naturally lead to the question of a role for synchronized oscillations at various frequencies between the midbrain and other brain regions. Dreyer et al. (2016) demonstrate that cocaine induces 0.5-Hz oscillations in dopamine release in the nucleus accumbens. Fujisawa and Buzsáki (2011) have demonstrated 4-Hz oscillatory activity in the VTA that couples with prefrontal oscillations. The authors suggest that midbrain dopamine may play a role in synchronizing this oscillatory activity across brain regions. Such oscillatory activity in the midbrain has been surprisingly little studied and could be introduced into the current hypothesis. Fourth, dopamine cells release multiple transmitters (Trudeau et al., 2014), including glutamate, GABA and sonic hedgehog (Gonzalez-Reyes et al., 2012), not addressed here. Our hypothesis is built around dopamine volume transmission. What role intrasynaptic neurotransmission at dopamine synapses may play is not clear. Interestingly, Fujisawa and Buzsáki (2011) suggest that glutamate release from dopamine terminals may play a role in regulating oscillatory synchronization. Although not incorporated into our hypothesis, this notion is certainly consistent with midbrain dopamine serving a role in coordinating distributed, parallel processing.

### Areas for development

Our hypothesis posits that dopamine cells comprise a layer in cascading learning. While dopamine cells exhibit synaptic plasticity, this plasticity and its regulation has not been as extensively characterized as, for example, corticostriatal plasticity. We suggest here that synaptic plasticity in dopamine cells is regulated by inhibitory GABA inputs from the striatum, analogous to how dopamine regulates corticostriatal plasticity. In this way, selective activity-dependent long-term potentiation of excitatory synapses would be gated by disinhibition of striatal inputs. Although there is evidence for this notion (Tan et al, 2010), it has not been exhaustively investigated and characterized. Synaptic plasticity within the midbrain has not, in general, figured prominently into theories of dopamine; knowledge of its mechanisms, regulation and function remain limited and underexplored. While dopamine has been suggested to provide an instructional signal to the striatum, we suggest that the striatum in turn provides an instructional signal to the midbrain. In this construal, this striatal teaching signal, the likelihood, gates plasticity at excitatory synapses, modifying transmission of excitatory drive, the prior, from primary processing, in future encounters.

Another area for exploration is how different inputs are actually integrated at a cellular level. Our Bayesian construal would on first glance suggest that inhibitory inputs from the striatum and excitatory inputs from distributed afferents should be multiplicative, but this is not exactly right. The likelihood, P(I | E)/P(I) is not computed in the midbrain where excitatory activity, P(E) is multiplied by P(I | E) and both quantities divided by P(I). Rather, in our construal, the likelihood is computed in the striatum and delivered as a single quantity of disinhibition to the midbrain; mathematically, P(E) is not divided directly by P(I). The question might be whether P(E) + P(I | E)/P(I), likely a more accurate rendering, is functionally equivalent. Using cortical activity as an example, if specific cortical activity (say that encoding a cue light) contributes afferent drive to both midbrain, i.e., P(E) and the striatum, i.e., P(I | E), could an increase in this cortical activity transmitted to the midbrain both directly and via disinhibition be multiplicative in the degree to which that specific cortical activity drives midbrain dopamine? The answer is unknown. Demonstrating how mathematical operations comprising formal theories are implemented in neural machinery is a continuing challenge (Potjans et al., 2009, 2011).

Finally, our initial description of the hypothesis provided here requires more formal theoretical elaboration. However, doing so entails rethinking what a normative description of dopamine function might mean if its primary role is construed as mediating coordination across distributed processing rather than signaling value per se. As long as dopamine is viewed in some fashion as the reward transmitter, computational approaches can build formal algorithms whose functional goal is maximizing value. Our hypothesis posits “maximizing value” as a distal goal mediated by multiple neural substrates where the proximal problem is getting those substrates to function in a coordinated manner to achieve maximal value. If dopamine is construed as a solution to that proximal problem of coordination, then thinking through formal models of this idea requires developing notions on how “coordination” is maximized or even quantified and evaluated. We believe our Bayesian construal of dopamine and notion of cascading, distributed learning offers some initial fodder for more formal efforts.

## Conclusion

A good hypothesis should provoke novel investigations and generate deeper understanding, whether ultimately proven true or not. The prediction error hypothesis makes clear, simple predictions: that phasic dopamine signaling will correspond to discrepancies in expected and actual outcomes. Nonetheless, twenty years later we are still testing this hypothesis (Watabe-Uchida et al., 2017), which has proven to be a rich driver of experimental investigation giving rise to a deeper, more complex understanding of midbrain dopamine (Eshel et al., 2015; Hamid et al., 2016; Langdon et al., 2018), although the extent to which it is correct continues to be subject to debate (Berke, 2018).

Various theories of dopamine function have been proposed. Data have accumulated supporting each of these theories and, strictly speaking, falsifying each other. If a value instead of error signal is observed, then strictly speaking the prediction error hypothesis cannot be a complete account. The taste for pitting these different accounts of dopamine against each other seems to be waning, with a growing appreciation that each likely captures some aspect of dopamine signaling. Increasingly, the most pressing question seems to be how to conceive of a framework where these different theories can be integrated rather than viewed as competing accounts.

By shifting from a presupposition that dopamine fundamentally signals value information in some fashion, the reward transmitter, to positing that dopamine plays a role in mediating coordination across distributed, parallel processing, the hypothesis outlined here provides an alternative perspective in thinking about how to assimilate and interpret the diverse data that have accumulated for decades on dopamine. Moreover, it suggests new avenues for both theoretical and empirical exploration and model development. This is particularly true in light of data emerging in recent years showing much greater richness and complexity to dopamine signals, as well as its connectivity, architecture and physiology, than originally imagined. As it becomes increasingly difficult to shoehorn this apparent complexity into one or another extant theories, the need for alternative frameworks, perspectives and accounts may grow. Although only an initial description, we offer the current hypothesis in the spirit of this need for new perspectives.

## Synthesis

Reviewing Editor: Lorna Role, NINDS Decisions are customarily a result of the Reviewing Editor and the peer reviewers coming together and discussing their recommendations until a consensus is reached. When revisions are invited, a fact-based synthesis statement explaining their decision and outlining what is needed to prepare a revision will be listed below. The following reviewer(s) agreed to reveal their identity: GIANCARLO LACAMERA, Niraj Desai.

The reviewers (there have now been 3 plus the reviewing editor) all concur that this manuscript contains interesting and thought-provoking ideas related to understanding how dopamine functions.

Despite the fact that the ideas are speculative and not fully formed, the consensus is that it would benefit the neuroscience community if they were published, but not as a theory paper per se.

***The summary recommendation on this R1 is that the paper will only be reconsidered for publication in eNEURO as a Perspective/ Commentary piece. ***

The reviewers strongly agreed with one another that the paper should not be reconsidered for publication as a research paper in the “theory & neural computation” section, as the original and R1 were submitted.

The reviewers feel that the paper could be a very good perspective paper which puts forward these good, new ideas about the role of dopamine, but that the authors need to do so in a considerably shorter document and in a manner that is as clear, concise and simple as possible to have appeal to the broader e NEURO readership.

The comments fall into 2 general domains:

A. Addressing a few issues that were raised about this paper in the prior review but that were unchanged in the revision

and

B. Specific suggestions as to how to trim the paper down and focus the discussion, to optimize clarity.

We recognize that rewriting and resubmission of the paper as a perspective would require major revision but the paper must be shorter and focused on conveying the basic ideas in a clear and simple manner.

A. Issues that were raised about this paper in the prior review but that are unchanged in the revision.

1. Re: Theory vs. perspective/ideas

Even though we cannot require neuroscience to have the same standard as physics or chemistry when it comes to theory, there are an increasing number of ‘true’ neuroscience theory papers that do abide by stricter rules.

While the reviewers agree with the authors that previous frameworks on DA functions such as those of Berridge and Robinson or those of Robbins and Everett have been very influential, they do not agree that an idea, to be influential, must be a theory. S/he states: “the Hebbian paradigm has been such an influential paradigm, but it didn't become a proper theory until it was developed on a more quantitative basis -- at which points its problems, such as instability, also became apparent.”

In sum: although the community does welcome new ideas,it is not required that those ideas are put forth as theories, per se. Hence the suggestion to reframe this as a commentary/perspective piece.

2. Re: the biological plausibility of the Bayesian construct, as put forward by the authors of this manuscript.

One of the reviews reiterated that the issues previously raised with respect to the biological plausibility of the Bayesian construct is not the lack of anatomical evidence for quantities such as P(E|I) etc; it is the lack of a credible implementation in neural terms. S/he states “Neurons are good at adding up inputs, not at multiplying them as required by Bayes rule. A good explanation of how neural circuits can implement Bayesian computations is lacking -- not just in this paper” -In the reviewers opinion “it is a necessary ingredient to give credence to the hypothesis that the brain performs some form of Bayesian inference” While s/he “doesn't want to force the authors to solve this very difficult problem, [s/he] warns that making Bayesian claims in these general terms although easy to do, does not gain us much”.

To be more concrete, the reviewers ask that you present your Bayesian idea in more hypothetical terms (more like it would be in a grant proposal than in a paper). You can then argue for the anatomical considerations, but you should specify that it would be necessary to demonstrate in an anatomically compatible circuit-based model (by others, not in this paper) that E and I neurons in that circuit can be used to perform something akin to Bayesian inference. Then, assuming that that is possible, you can discuss how to use that inference for your ‘distributed consensus’ idea.

3. Related to the example of temporal difference learning.

The reviewer argues that “it [ temporal difference learning] is a proper theory, but just like Bayesian accounts, it lacks a convincing neural implementation. In fact, the only attempt [s/he] knows of “to implement TD learning in biologically plausible circuitry, is in two papers by Potjans, Diesmann and Morrison (Neural computational 21:301, 2009 and Plos Computational Biology 7(5):e1001133, 2011).” S/he further comments that “It is not much, and there's room for improvement, but at least it's a start. It would probably be a good idea to cite these 2 papers as examples of what the field should be attempting to do”

4. Related to the need to trim the paper, as well as to frame it as a perspective piece.

S/he had “ hoped the paper would be trimmed .. but in fact, it has become bigger.” This reviewer also noted that the figures are not helpful in illustrating the concepts expressed in the paper” The reviewer recommends that the authors do their best to cut down the number of figures and work on making the figures that they do choose to keep much clearer with respect to how clear they are. It is suggested that you should show the figures to other neuroscientists (not co-authors) and see what recommendations they can make toward enhancing clarity.

B. Specific suggestions as to how to trim the paper down and focus the discussion, to optimize clarity.

Overall, the reviewers felt that in articulating a general theory of dopamine the authors have been rather vague. Still, the proposals (speculations) are seen as creative and stimulating and the reviewers believe it will provoke valuable debate if the article is better focused. But to spark a debate - the article must be read. Our consensus is that the present manuscript would not be read through because it is too wordy and disorganized.

The specific recommendations include

1) re: LESS IS MORE:

The author(s) of this manuscript are good writer(s) but - as is often the case with good writers - the document would benefit from the services of a pitiless editor - someone who would slash the word count, tone down the stylistic flourishes (such as the overuse of italics for emphasis) and minimize the number of repetitions (e.g. the reader is reminded at regular intervals that the present interpretation is different than what has gone before).

There are several sections of this paper that could be cut down or eliminated altogether ( the latter is up to you).

(i) The first ten pages or so constitute a review of ideas about dopamine. It's a nice review, but there have been many, many previous dopamine reviews and this isn't supposed to be a Review paper. This section should be cut down to 1/4 of its current size. One might also try to guide the reader focus this background on the specific issues that lead them to the authors own ideas (other than by indicating that *something* is wrong with conventional ideas).

(ii) Much of the latter part of the paper is devoted to explicating the theory's limitations and the things (the Schultz experiments) the theory doesn't obviously capture. But if one thinks of this paper as a Perspective paper offering up some interesting but incomplete ideas - rather than a grand unified theory - than these limitations and omissions require no comment.

(iii) Prefrontal cortex. The reviewers suggest that elimination of this part of the paper may help with focus -- butthis is up to the authors

2) Re discussion of dopamine in basal ganglia:

While the reviewers felt that for the most part the paper should be shorter and more concise, this is one section that needs elaboration. The striatum plays a big part in the model (namely, the ‘I’ of P(E/I)). Yet, the discussion of how the striatum contributes is cursory and rather confusing. (at least 2 of us read those sections repeatedly and still didn't get how the authors think the BGS operates). This section also includes many new and not so meaningful terms (discriminative integration” and ‘Selective integrative’, ‘consensus integration’ and ‘distributed coherence’). It would be more accessible if the authors eliminate these terms and simply describe more clearly what they really mean. There is no doubt that the interaction of basal ganglia and midbrain is absolutely crucial to their perspective being broadly useful. It's worth the time and effort to be sure this is clearly stated.

In addition, the authors use the terms coherence and bootstrapping in colloquial ways. For systems neuroscientists, these terms have specific technical meanings in the context of oscillations and statistics, respectively. The authors should use other words.

3) Re: HYPOTHESIS TESTS:

In response to the first round of reviews, the authors added a section on tests of their hypothesis. The part that was particularly confusing is the first (lines 945-954). If this is to be included in the perspective, it would be helpful for the authors to reword that section, so that it's clearer how that is a test.

## Author Response

The reviewers (there have now been 3 plus the reviewing editor) all concur that this manuscript contains interesting and thought-provoking ideas related to understanding how dopamine functions.

Despite the fact that the ideas are speculative and not fully formed, the consensus is that it would benefit the neuroscience community if they were published, but not as a theory paper per se.

Thank you.

***The summary recommendation on this R1 is that the paper will only be reconsidered for publication in eNEURO as a Perspective/ Commentary piece. *** The reviewers strongly agreed with one another that the paper should not be reconsidered for publication as a research paper in the “theory & neural computation” section, as the original and R1 were submitted.

The reviewers feel that the paper could be a very good perspective paper which puts forward these good, new ideas about the role of dopamine, but that the authors need to do so in a considerably shorter document and in a manner that is as clear, concise and simple as possible to have appeal to the broader e NEURO readership.

We have revised the manuscript as a perspective paper and reduced it by nearly half. We have endeavored to make it more readable and focused on developing key ideas rather than nit-picking or ‘arguing in advance’ with potential objections. We wish to thank reviewers for their persistence in this. While initially our position was that this should be a theory paper, having done this revision we now agree with the reviewers whole-heartedly in their suggestion. Frankly, it was liberating to rewrite the manuscript as a Perspectives paper. As a theory paper, even absent mathematical formalities, we felt compelled to fill in every detail and answer every possible objection. . . which resulted in an excessively long paper that could at times become confusing and ‘go down the rabbit hole,’ as it were. Despite this, there still remained more to be said and developed. And all of this, in a sense, premature as our goal is to put out the main, overall idea and propose a shift from dopamine mediating value to dopamine mediating coordination across distributed processing. We feel that the perspective format allows us a little more leeway to sketch out an initial description of our hypothesis in a more readable and, ironically, convincing way and to get it out to the community for comment and discussion and further future development, by us and possibly others.

We recognize that rewriting and resubmission of the paper as a perspective would require major revision but the paper must be shorter and focused on conveying the basic ideas in a clear and simple manner.

We agree and hope we have been successful in this regard.

1. Re: Theory vs. perspective/ideas

Even though we cannot require neuroscience to have the same standard as physics or chemistry when it comes to theory, there are an increasing number of ‘true’ neuroscience theory papers that do abide by stricter rules.

While the reviewers agree with the authors that previous frameworks on DA functions such as those of Berridge and Robinson or those of Robbins and Everett have been very influential, they do not agree that an idea, to be influential, must be a theory. S/he states: “the Hebbian paradigm has been such an influential paradigm, but it didn't become a proper theory until it was developed on a more quantitative basis -- at which points its problems, such as instability, also became apparent.”

In sum: although the community does welcome new ideas,it is not required that those ideas are put forth as theories, per se. Hence the suggestion to reframe this as a commentary/perspective piece.

Noted. We appreciate the reviewers' viewpoint and are happy to submit as a Perspective paper.

2. Re: the biological plausibility of the Bayesian construct, as put forward by the authors of this manuscript.

One of the reviews reiterated that the issues previously raised with respect to the biological plausibility of the Bayesian construct is not the lack of anatomical evidence for quantities such as P(E|I) etc; it is the lack of a credible implementation in neural terms. S/he states “Neurons are good at adding up inputs, not at multiplying them as required by Bayes rule. A good explanation of how neural circuits can implement Bayesian computations is lacking -- not just in this paper” -In the reviewers opinion “it is a necessary ingredient to give credence to the hypothesis that the brain performs

some form of Bayesian inference” While s/he “doesn't want to force the authors to solve this very difficult problem, [s/he] warns that making Bayesian claims in these general terms although easy to do, does not gain us much”.

To be more concrete, the reviewers ask that you present your Bayesian idea in more hypothetical terms (more like it would be in a grant proposal than in a paper). You can then argue for the anatomical considerations, but you should specify that it would be necessary to demonstrate in an anatomically compatible circuit-based model (by others, not in this paper) that E and I neurons in that circuit can be used to perform something akin to Bayesian inference. Then, assuming that that is possible, you can discuss how to use that inference for your ‘distributed consensus’ idea.

This clarification of the objection to our Bayesian construal was helpful, i.e., that it is the neural implementation that is in question, not connectivity. In the revised Perspective paper, we present this as a hypothesis and note that questions of neural implementation remain. In fact, we note more specifically that in our construal, the ‘E’ in P(E) and P(I|E) is not actually the same ‘E’ and include a brief discussion of how our Bayesian proposal does not exactly match the mathematical formulation. As the reviewers note, matching equation to neurons is a fundamental challenge for almost all formal theories, Bayesian or not, which we cannot solve. That said, we think it strengthens the paper to note this problem explicitly.

3. Related to the example of temporal difference learning.

The reviewer argues that “it [ temporal difference learning] is a proper theory, but just like Bayesian accounts, it lacks a convincing neural implementation. In fact, the only attempt [s/he] knows of “to implement TD learning in biologically plausible circuitry, is in two papers by Potjans, Diesmann and Morrison (Neural computational 21:301, 2009 and Plos Computational Biology 7(5):e1001133, 2011).” S/he further comments that “It is not much, and there's room for improvement, but at least it's a start. It would probably be a good idea to cite these 2 papers as examples of what the field should be attempting to do”

Thank you for highlighting these two papers. We have now added reference to them in alluding to the challenge of demonstrating plausible neural implementations of theories. As an aside, we believe that in moving forward and developing the hypothesis we are describing here, it is precisely this sort of spike-based representations of formal expressions that will be useful in developing our hypothesis further. We agree, more of this in the theoretical

4. Related to the need to trim the paper, as well as to frame it as a perspective piece.

S/he had “ hoped the paper would be trimmed .. but in fact, it has become bigger.” This reviewer also noted that the figures are not helpful in illustrating the concepts expressed in the paper” The reviewer recommends that the authors do their best to cut down the number of figures and work on making the figures that they do choose to keep much clearer with respect to how clear they are. It is suggested that you should show the figures to other neuroscientists (not co-authors) and see what recommendations they can make toward enhancing clarity.

We have now cut it nearly in half. Moreover, what remains should be considerably more readable and thus make the subjective feel of reading it even shorter.

We have removed one figure. We still struggle with the reviewers' unhappiness with our figures. They are intended to convey simple concepts. We have discussed our figures with colleagues. We have completely revised one figure to make it more simple (less lines swooping all over) and linear (the PFC-BG-DA figure) and with the main ‘excitatory-inhibitory axes’ figure we have added a second panel that includes a little more anatomical detail (without cluttering too much), including sketching in the basal ganglia loops. We have made some additional minor changes in other figures to improve. We hope these changes, in concert with textual changes, will enhance the usefulness of the figures in conveying basic ideas.

B. Specific suggestions as to how to trim the paper down and focus the discussion, to optimize clarity.

Overall, the reviewers felt that in articulating a general theory of dopamine the authors have been rather vague. Still, the proposals (speculations) are seen as creative and stimulating and the reviewers believe it will provoke valuable debate if the article is better focused. But to spark a debate - the article must be read. Our consensus is that the present manuscript would not be read through because it is too wordy and disorganized.

The specific recommendations include

1) re: LESS IS MORE:

The author(s) of this manuscript are good writer(s) but - as is often the case with good writers - the document would

benefit from the services of a pitiless editor - someone who would slash the word count, tone down the stylistic flourishes (such as the overuse of italics for emphasis) and minimize the number of repetitions (e.g. the reader is reminded at regular intervals that the present interpretation is different than what has gone before).

We have slashed without pity. A product of the slashing is less repetition. . . which often arose because the text in the prior manuscript would stray so far from the main idea it felt the main idea needed to be repeated. This is not a problem in the revised, perspective piece, which flows along a more straightforward path from point to point.

There are several sections of this paper that could be cut down or eliminated altogether ( the latter is up to you).

(i) The first ten pages or so constitute a review of ideas about dopamine. It's a nice review, but there have been many, many previous dopamine reviews and this isn't supposed to be a Review paper. This section should be cut down to 1/4 of its current size. One might also try to guide the reader focus this background on the specific issues that lead them to the authors own ideas (other than by indicating that *something* is wrong with conventional ideas).

We have cut the review aspects of the manuscript greatly. For example, the detailing of different prior theories, which previously was an entire section, was collapsed to one brief paragraph and combined with two other equally condensed sections. In general, in the revision we found we could reduce and combine 2 or more sections, often reducing by 30-50%. We focus our ‘review’ text on highlighting ideas and data central to the hypothesis we are describing. Further, as the reviewers suggest, we spend less time in the revised manuscript discussion ‘what is wrong’ with conventional ideas and focus more on just describing our proposed alternative perspective.

(ii) Much of the latter part of the paper is devoted to explicating the theory's limitations and the things (the Schultz experiments) the theory doesn't obviously capture. But if one thinks of this paper as a Perspective paper offering up some interesting but incomplete ideas - rather than a grand unified theory - than these limitations and omissions require no comment.

This was perhaps the most liberating aspect of shifting to a perspective paper. In our prior manuscripts, we were trying to cross all the t's and dot all the i's and address any objection, which led us down a rabbit hole. We have eliminated most of this. We did feel the need to retain a brief comment about

the two cases in which any reasonable reader in the dopamine field would say ‘but wait. . . ’ i.e., the loss of DA for well known rewards and the activation of DA in response to novelty. However, we touch on these very briefly, literally reducing what was previously several pages to just a couple of paragraphs. In addition, rather than enumerate tests of the hypothesis, which honestly readers can discern for themselves, we created a section that covers limitations and areas to explore. i.e., because this is a perspective paper, there is much more that could be said/developed but is not, we kept a small section to highlight some of these. Again, the reduction here is draconian, from several pages to a few paragraphs.

(iii) Prefrontal cortex. The reviewers suggest that elimination of this part of the paper may help with focus -- butthis is up to the authors

We went back and forth on this. While it is, in a sense a distraction, it also places the hypothesis we are describing into a broader context, which we feel is important. We compromised in that we retained this but touch on it only very briefly. Instead of serving as a ‘grand conclusion’ we now put it in the section where we introduce the problem of coordination of distributed processing. . . and then we move on and continue our focus on dopamine and the basal ganglia. We also originally removed the figure but opted to revise it to make it more comprehensible and to retain it. We did this specifically because a picture is worth a thousand words. We believe this ‘axis of agency’ notion is both defensible and an intriguing idea and we would like it to be included and communicated. We feel this is particularly important as there is often a gap between those that study PFC/cortical function and those that study the basal ganglia where each field sort of brackets off the other (e.g., action selection in basal ganglia, then what is cortex doing?). We feel there is value in sketching a broader scheme proposing a specific functional relationship of these two areas in service to some larger, overriding problem to be solved. Even if only briefly.

2) Re discussion of dopamine in basal ganglia:

While the reviewers felt that for the most part the paper should be shorter and more concise, this is one section that needs elaboration. The striatum plays a big part in the model (namely, the ‘I’ of P(E/I)). Yet, the discussion of how the striatum contributes is cursory and rather confusing. (at least 2 of us read those sections repeatedly and still didn't get how the authors think the BGS operates). This section also includes many new and not so meaningful terms (discriminative integration” and “Selective integrative”, “consensus integration” and “distributed coherence”). It would be more accessible if the authors eliminate these terms and simply describe more clearly what they really mean. There is no doubt

that the interaction of basal ganglia and midbrain is absolutely crucial to their perspective being broadly useful. It's worth the time and effort to be sure this is clearly stated.

We have revised this section and attempted to convey the main idea better, including adding some substructure to the section. While we retain the terms the reviewers find confusing, we recognize that we were using those terms as if they were self-explanatory, expecting those words to do the work of explaining. In the revision, we explain very clearly what those words mean (i.e., why ‘integrative’ why ‘selective’ and so on), using those words to encapsulate ideas we now explain. We felt we had a thin line to walk here. A full explication of our conceptualization of the basal ganglia could be (actually, will be) a paper unto itself. At the same time, we appreciate that our prior rendering was too cursory and too wordy. We aimed in the revision to be more clear and focused.

In addition, the authors use the terms coherence and bootstrapping in colloquial ways. For systems neuroscientists, these terms have specific technical meanings in the context of oscillations and statistics, respectively. The authors should use other words.

We agree with reviewers regarding the word ‘coherence’ and have removed it and use instead ‘coordination.’ Regarding bootstrapping, we do not feel that statisticians get to own this word. However, in the general spirit of stripping down and brutally excising unnecessary text, we have entirely eliminated the discussion of bootstrapping from the revision, making this a moot point. We believe the idea we were conveying with the ‘bootstrapping’ sections is implicit and, moreover, applicable to basically any theory of DA. In short, it did not significantly contribute to conveying our main ideas and so was mercilessly removed altogether.

3) Re: HYPOTHESIS TESTS:

In response to the first round of reviews, the authors added a section on tests of their hypothesis. The part that was particularly confusing is the first (lines 945-954). If this is to be included in the perspective, it would be helpful for the authors to reword that section, so that it's clearer how that is a test.

We have removed the tests. As a Perspective, we view this as ‘pre-theory’ or ‘theory in emergence.’ To be honest, readers can easily discern tests themselves and there is little need for us to occupy space enumerating them. We note this with a couple of brief examples of the ‘obvious’ tests (literally a couple of sentences). Instead, we opt to highlight areas that we did not

incorporate into our hypothesis but which are interesting, relevant and could/should be considered at some point, as well as areas we think merit more exploration, both of which seem more appropriate to a perspective piece. It is also in this section that we briefly touch upon implementational problems/questions with our Bayesian construal. To be honest, we felt the ‘tests’ section in our prior manuscript was becoming increasingly tortured. We are grateful for the opportunity to simply highlight some areas for further thought and development, which seems more appropriate for a perspective piece.

In closing, we want to reiterate our appreciation for the reviewers. While initially we objected to their insistence that we reframe this as a perspective piece, in retrospect the reviewers were 110% right. We feel the manuscript is much better and the spirit of the prose and presentation more appropriate to the level of development of the ideas presented. Thank you.
